# Benefits of bone marrow-derived mesenchymal stem cells primed with estradiol in alleviating collagen-induced arthritis

**DOI:** 10.22038/IJBMS.2023.68112.14882

**Published:** 2023-04

**Authors:** Monireh Jahantigh, Seyyed Meysam Abtahi Froushani, Nahideh Afzale Ahangaran

**Affiliations:** 1 Department of Microbiology, Faculty of Veterinary Medicine, Urmia University, Urmia, Iran

**Keywords:** Estradiol, Inflammation, Mesenchymal stem cells, Rheumatoid arthritis, Wistar rat

## Abstract

**Objective(s):**

To investigate the effects of the oestradiol (ES) pulsed bone marrow-derived mesenchymal stem cells (BM-MSC) to treat adjuvant-induced arthritis in Wistar rats.

**Materials and Methods:**

BM-MSCs were pulsed with ES (0, 10,100, and 1000 nM) for 24 hr. RA was induced by collagen and Freund’s Complete Adjuvant into the base of the tail of Wistar rats.

**Results:**

The least effective concentration of ES that can promote potent anti-inflammatory properties in the MSC population is 100 nM. At this concentration, ES increases the inhibition of the polyclonal T lymphocyte proliferation, production of IDO, IL-10, Nitric oxide, and TGF-β, and expression of CXCR4 and CCR2 mRNA in the MSC population. Accordingly, the RA rats were treated with 2×106 MSCs or ES-pulsed MSCs (100 nM) on day 10 when all animals had developed signs of RA. ES-pulsed BM-MSCs reduced the severity of RA more profoundly than treatment with BM-MScs alone. The ability of ES-pulsed BM-MSCs to reduce symptoms and RA markers like CRP, RF, and nitric oxide was comparable to that of prednisolone. Prednisolone was more successful in reducing inflammatory cytokines than treatment with ES-pulsed BM-MSCs. ES-pulsed BM-MSCs were more successful in increasing anti-inflammatory cytokines than treatment with Prednisolone. The ability of ES-pulsed BM-MSCs to decrease the level of nitric oxide was comparable to that of prednisolone.

**Conclusion:**

ES-pulsed BM-MSCs may be a helpful strategy in RA control.

## Introduction

Rheumatoid arthritis (RA) is an inflammatory disease characterized by joint inflammation and synovitis, swelling, autoantibody production, bone dysfunction, and cartilage degradation (1, 2). The main features of RA are inflammatory cell infiltration in the joints, increased concentration of inflammatory factors, and invasion of adjacent cartilage, all of which cause bone erosion and cartilage tissue damage (2). The etiology of the disease is not known. However, it might be an autoimmune disease owing to the production of autoantibodies against citrulline proteins (3, 4). 

Several conventional agents are administrated to alleviate RA progression, such as steroidal and nonsteroidal anti-inflammatory drugs, disease-modifying anti-rheumatic drugs (DMARDs), and novel biological therapeutic agents. Despite advances in RA control, no drug can completely cure the disease. In addition, most of these drugs have dangerous side effects (3, 5, 6). In this regard, much attention has been paid to the therapeutic potential of mesenchymal stem cells (MSCs). MSCs, like bone marrow-derived mesenchymal stem cells (BM-MSCs), are found in the bone marrow and differentiated into adipocytes, osteoblasts, and chondrocytes. Due to their high immunomodulatory and regenerative potentials, these cells could be considered a practical approach to controlling autoimmune diseases such as rheumatoid arthritis (7). Administration of the BM-MSCs helped restore damaged cartilage and decreased synovial inflammation (8). MSC therapy is a unique strategy for migrating transplanted cells toward injured targets. However, a small percentage of the transplanted cells reach the target tissues (9-11). The increased number of MSCs in the injury sites may improve the efficacy of MSC transplantation (9). The presence of estrogen receptors (ERα and ERβ) and their responsive elements within MSCs suggest that estrogen is involved in modulating the function of these cells. The studies have reported that the differentiation, migration, and immune regulatory function of MSCs depend on multiple nuclear receptors such as nuclear steroid receptors (12, 13). Many studies have shown that estrogen derivatives, as well as pregnancy, play a protective role in rheumatoid arthritis by directly modulating immune responses (14). On the other hand, the use of estradiol (17β-estradiol) has a pivotal role in proliferation, differentiation, and maturation of hematopoietic progenitor cells expressing estrogen receptor α (ERα) (15, 16). It has been reported that treating MSCs with 17β-estradiol improved the viability and function of neutrophils (7). Some studies have investigated the treatment of BM-MSCs with estradiol for the treatment of diabetes (9), experimental autoimmune encephalomyelitis (17), and ulcerative colitis. However, the efficiency of ES-pulsed MSCs for the treatment of RA has not been reported. Thus, this study investigates the role of the BM-MSCs primed with estradiol (ES) in alleviating collagen-induced arthritis in Wistar rats.

## Materials and Methods

***Materials***

RPMI 1640, phosphate-buffered saline, fetal bovine serum,

and penicillin-streptomycin were prepared by Biowest Company. Dimethyl sulfoxide, 3,3′,5,5′-Tetramethylbenzidine, Freund’s Complete Adjuvant (FCA), estradiol, and collagen II were procured from Sigma Aldrich Company (USA). Prednisolone was purchased from Aburaihan company (Tehran-Iran). Cytokines were assessed by Commercial kits prepared by Peprotech company (USA), and other kits were purchased from Sigma Aldrich company (USA).

***Isolation and proliferation of MSCs***

The BM-MSCs were isolated and proliferated, as reported previously (18). Briefly, BM-MSCs were obtained from tibias and femurs of Wistar rats (6 weeks old). They were washed twice and centrifuged at 1200 rpm for 5 min in PBS, and cells were plated in tissue-culture flasks at the concentrations of 0.3 to 0.4×10^6 ^cells/cm^2^. DMEM medium was supplemented with 15% fetal bovine serum, and cells were incubated in a humidified incubator with 5% CO_2_ at 37 °C. After four days, the culture mediums were collected, centrifuged, and cells were seeded. Cells were trypsinized using Trypsin/EDTA, counted, and passed at 1:3 ratios (about 1.5 × 10^6^ cell/75-cm^2^ flask). The MSCs were incubated upon 70% confluency, trypsinized, collected, and used for the following experiments. Cells in the sediment were soaked in 1 ml sterilized PBS and formaldehyde (40 µl) was added to the mixture and incubated at 37 ºC for 10 min. The investigation of phenotyping of the cultured cells was conducted as reported by Jones *et al*. (19). 

***Immunophenotype of MSCs***

To characterize MSCs, these cells at passage three were stained with a fluorescently labeled monoclonal antibody (anti-rat CD29 (Integrin b chain; Ha2/5; FITC), CD90-PCY5 (Thy-1/Thy-1.1-FITC), and CD45-FITC) as described formerly (20). The stained cells were monitored immediately on a DAKO flow cytometer (Partec, Germany).

***Estradiol treatment of MSCS***

Cells at the third passage were treated with 0, 10, 100, and 1000 nM 17β-estradiol for 24 hr. The medium was gathered, and the cells were washed three times with PBS. 

Evaluation of the immunoregulatory potential of MSCs:

To evaluate the potency of MSCs in inhibiting lymphocyte proliferation, spleens were aseptically isolated from 3 Wistar rats. MSCs were plated at 4×10^3^ cells/cm^2^. After a short adherence period, splenocytes were incubated with the plated MCs (10 splenocytes to 1 MSC) in a trans-well system (0.4-mm pore size membrane, eight-well strip, Nunc). For mitogenic stimulation, splenocytes were stimulated with phytohemagglutinin (5 μg/ml). After five days, the solenocytes were pulsed with 20 μl of the MTT solution (final concentration: 5 mg/ml). To dissolve the formazan crystal, 150 ml DMSO was added to plates after four hours. The plates were shaken vigorously. The optical density (OD) at 550 nm was monitored by a microplate reader (Dynatec, Dockendorf, Germany). The results were expressed as the proliferation index (PI) on the MTT assay calculated according to the ratio of OD_550_ of stimulated cells with PHA to OD_550_ of non-stimulated cells.

For *in vitro* examination, the isolated conditioned media were filtered through a 0.22 mm membrane, and the levels of TGF-β and IL-10 in conditioned media were monitored using commercial ELISA kits (Peprotech Company, USA). The levels of nitric oxide (NO) in conditioned media were determined by the Griess method (4). The biological activity of Indoleamine 2, 3-dioxygenase (IDO) was evaluated by monitoring the level of kynurenine in the isolated conditioned media (20). 

In original research, the pivotal importance of CCR2 and CXCR4 chemokine receptors has been shown in migrating mesenchymal stem cells toward damaged tissues (9). To analyze the mRNA expression of CXCR4 and CCR2, total RNA was extracted from the MScs using the Trizol reagent. Then, the complementary DNA was synthesized by isolated mRNA. PCR amplification was run in triplicate by the SYBR-Green kit (Parstous, Iran) according to the manufacturer’s guidelines. The sequence of primers was selected based on a previous article (9). The primers are shown in Table 1. Cyclic conditions were conducted in an Eppendorf Master cycler (Hamburg, Germany). In the case of CXCR4, each cycle is composed of 5 min at 94 ^°^C, followed by 40 cycles of 30 sec at 94 ^º^C, 1 min at 52 ^º^C, and a final step of 5 min at 72 ^º^C. In the case of CCR2, each cycle is composed of 5 min at 94 ^°^C, 30 sec at 94 ^°^C, 1 min at 55 ^°^C, and 5 min at 72 ^°^C. Each cycle of β-actin consisted of 5 min at 94 ^°^C, 1 min at 56 ^°^C, and 1 min at 72 ^º^C. PCR progressed up to 35 cycles. All target genes’ findings were expressed as relative fold change (RFC) from the control group (estradiol concentration = 0) values. 

***Induction and evaluation of the RA***

Animal studies were conducted under the ethical code “IR, UU.AEC. 476/PD3” issued by the Ethics Committee for Laboratory Animals of Urmia University. RA was induced as described by Brand *et al*. (21). Male Wistar rats (6–8 weeks old) were bought from the Pasteur Institute of Tehran, Iran. Animals were adapted to the environment for seven days. To induce RA, collagen II (2 mg/ml) was dissolved in acetic acid, homogenized overnight, and stored at -20 °C. Before administration, the collagen solution was mixed with 2.5 ml FCA and stored on ice. The emulsion was intradermally administrated using a syringe with 27–25 gauge at the base of the tail. The experimental rats were divided into five groups, each group comprising ten rats designated as: 1) Healthy rats included animals in which RA was not induced, 2) Negative control included the RA induced rats without the treatment, 3) Positive control included the RA induced rats that received daily treatment with 2 mg/kg of prednisolone, 4) BM-MSCs included the RA induced rats and treated with 2×10^6^ BM-MSCs (BM-MSCs), and finally 5) BM-MSCs+ES include rats induced with RA and treated with 2×10^6^ ES-pulsed BM-MSCs.

The intensity of RA was monitored by the following scoring system for each limb: 0=Normal paw; 1=Erythema of the toe; 2=Erythema and swelling of paws; 3=Swelling of the ankle; 4=Complete swelling of the whole leg and incapacity to bend it. The maximum arthritis score can be 16. The observations were done every morning during the investigation by three independent observers. The therapy was started on day 10 when all animals had developed a sign of RA. Treatments were performed intraperitoneally. The signs included inflammation, redness, and stiffness in joints. Animals were monitored until day 35 after induction.

***Serological evaluation***

At the end of the survey, blood samples were isolated from the hearts of experimental rats under deep anesthesia. Nitric oxide concentration was investigated as reported by Bryan *et al*. (22) and based on the Griess reaction method. Also, the serum concentration of MPO was assessed by an ELISA reader at 450 nm wavelength, as reported earlier (23). The serum concentrations of rheumatoid factor (RF) and C-Reactive Protein (CRP) were assessed as reported by the kit producer company.

***Ex vivo investigation of lymphocytes proliferation and cytokine profile***

At the end of the study, the rats were euthanized, and the spleens were isolated, aseptically. The spleen-to-body weight ratios were reported using the formula: Spleen Index= (spleen weight/body weight) ×100.

Next, spleens were aseptically ground in a five milliliter media culture of RPMI containing 10% FBS and then passed from a network with a size of 0.2 mm. To remove red blood cells, the samples were adjacent to ACK-RBC lysis buffer for 5 min. A splenocyte suspension (2×10^6^ cells/ml) was cultured in 6-well plates and primed with collagen II (50 µg/ml) for 72 hr. The culture supernatants were used to determine the levels of IL-6, IL-1β, IL-10, IL-17, TNF-α, and TGF-β using ELISA kits according to the manufacturer’s guidelines.

Furthermore, splenocytes were cultured in 96-well flat-bottomed plates (10^5^ cells/100 µl/well) and were primed with collagen II (50 µg/ml) for 72 hr or the medium alone, as control, for 72 hr. Afterward, each well was pulsed with 20 µl of MTT (3-(4,5-dimethylthiazol-2-yl)-2,5-diphenyltetrazolium) solution (5 mg/ml) for four hours. Then, the plates were centrifuged, and the supernatant was isolated. To dissolve formazan crystals, 150 µl of dimethyl sulfoxide was mixed into each well, and the plates were shaken vigorously. The absorbance of wells was monitored at 492 nm. The proliferation index was determined according to the ratio of the absorbance of the splenocytes pulsed with collagen II to the absorbance of non-pulsed cells.

***Data analysis***

The data were investigated for normality using the Kolmogorov-Smirnov test. Since the data have a normal distribution, One-Way-analysis was used. The data were reported as mean ± standard deviation. 

## Results

***Immunophenotype analysis of BM-MSCs***

**Figure 1:**
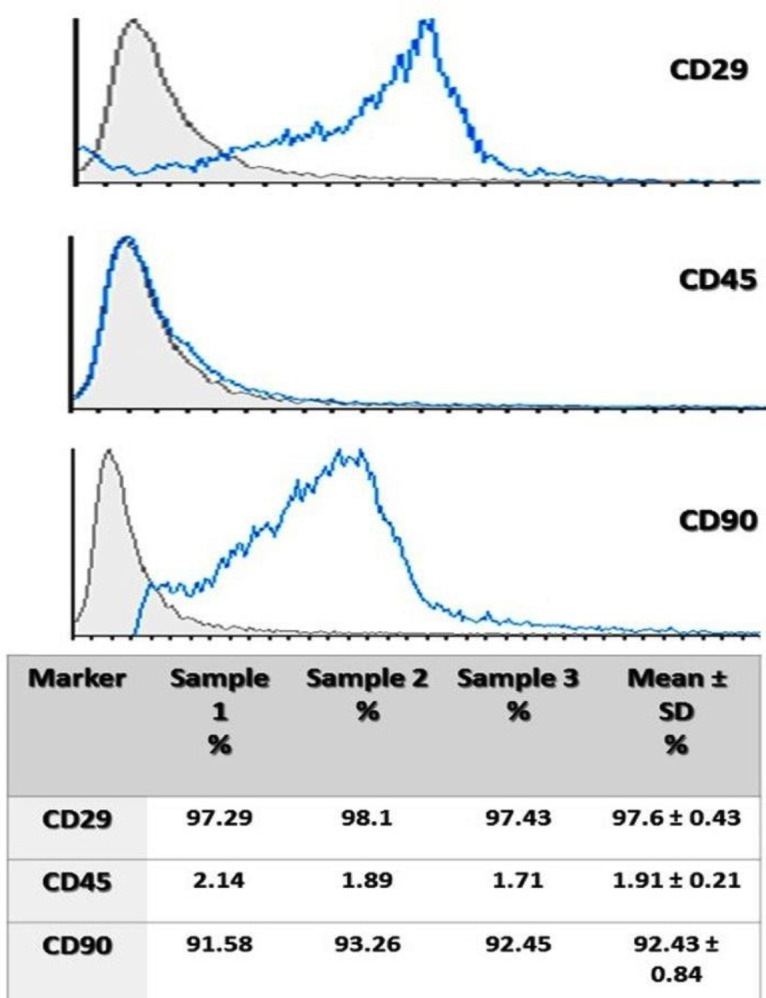
Immunophenotype evaluation of BM-MSCs. Flow cytometry analysis of BM-MSCs. BM-MSCs were negative for CD45 but positive for CD29 and CD90

The flow cytometry results showed that the BM-MSCs were positive for CD29 and CD90, two mesenchymal stem cell markers, and negative for CD45, an indicator of hematopoietic cells (Figure 1).

***Evaluation of the immunoregulatory potential of BM-MSCs in vitro***

**Figure 2:**
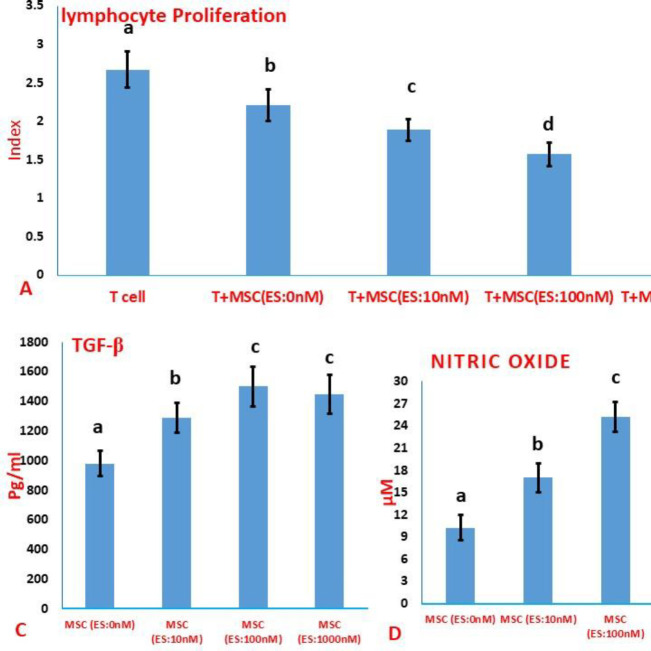
Effect of estradiol treatment on the immunoregulatory potential of MSCs. A) Assessment of the potency of MSCs in inhibiting lymphocyte proliferation. B-E) Evaluation of immunoregulatory factors in the conditioned medium of MSCs. Values were reported as mean ±SD. Different letters indicate a significant difference between groups (*P*<0.05)

In the presence of estradiol, the MSCs, ability to inhibit lymphocyte proliferation was strengthened. By increasing the estradiol concentration from 10 nM to 100 nM, the potential of MSCs to inhibit lymphocyte proliferation enhanced (Figure 2A). Nevertheless, there was no significant difference between the anti-proliferation potential of MSCs treated with 100 or 1000 nM of estradiol (Figure 2A). The levels of TGF-β, IL-10, NO, and IDO activity were significantly increased in conditioned media gathered from all ES-primed MSCs compared with conditioned media gathered from MSCs without treatment (Figure 2B-E). At the same time as the concentration of ES increased from 10 nM to 100 nM, the secretion of IL-10, TGF-β, and NO into the conditioned medium of MSCs significantly increased (Figure 2B-E). Regarding the production of IL-10 and TGF-β in the conditioned medium, there was no statistically significant difference between the MSCs pulsed with 100 nM and 1000 nM of ES (Figures 2B and C). Moreover, the NO level was significantly higher in the conditioned media isolated from estradiol-primed MSCs at 100 nM compared with the conditioned media gathered from MSCs pulsed with 0.1 mM of estradiol (Figure 2D). Although treatment with ES increased the IDO activity in the supernatant of MSCs, there was no significant difference between different groups treated with various concentrations of estradiol (Figure 2E).

**Figure 3:**
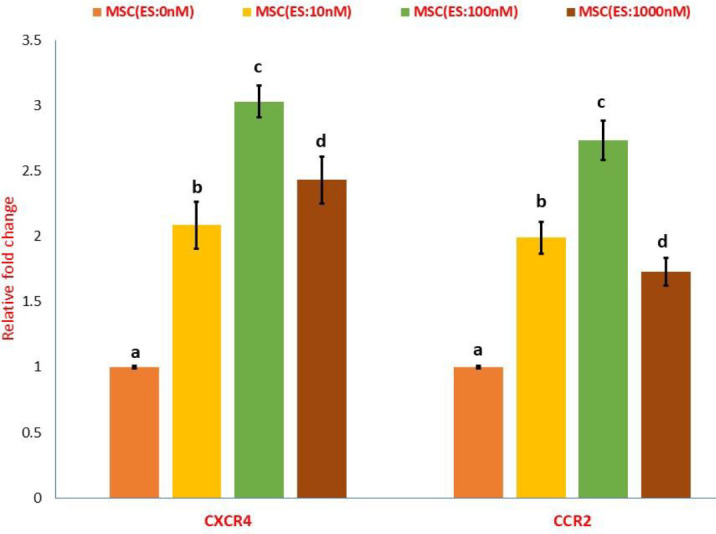
Effect of estradiol treatment on CXCR4 and CCR2 mRNA expression in MSCs. Results were presented as mean ±S.D. Different letters indicate a significant difference between groups (*P*<0.05)

To monitor the effects of estradiol on CCR2 and CXCR4 mRNA, reverse transcription PCR was performed. As depicted in Figure 3, all ES concentrations increased relative fold change (RFC) of both chemokine receptors in the MSCs population compared with un-pulsed MSCs. Increasing the estradiol concentration from 10 nM to 100 nM increased the expression of CCR2 and CXCR4 mRNA (Figure 3). However, the level of CCR2 and CXCR4 mRNA expression decreased significantly when the estradiol concentration increased from 100 to 1000 nM. The mRNA level of CCR2 was even lower than the mRNA expression of CCR2 induced by the concentration of 10 nM (Figure 3).

Based on *in vitro* findings, the least effective concentration of estradiol that can induce a potent anti-inflammatory phenotype in the MSCs was 100 nM. Therefore, this concatenation was chosen for *in vivo *studies.

***Results of using mesenchymal stem cells in rats with RA***

**Figure 4:**
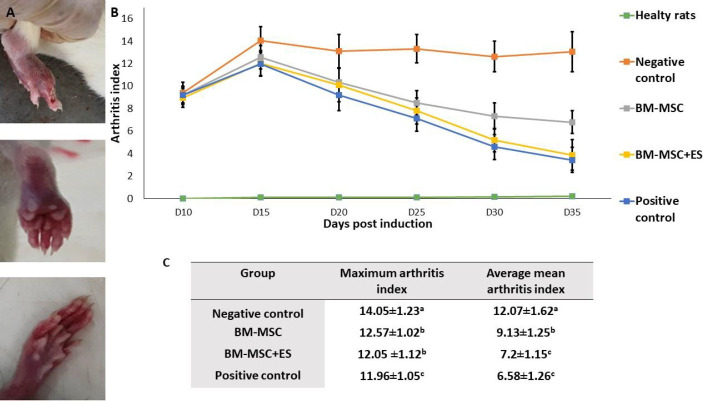
Evaluation of clinical features in RA rats. A) Appearance of swelling of the limb. B) Mean of arthritis index. C) Alteration of average mean arthritis index and maximum arthritis index. The *in vivo *results showed that the treatment with ES-pulsed BM-MSCs or prednisolone (positive control) reduced the severity of RA more profoundly than treatment with BM-MScs alone. Data were reported as mean ±S.D. Different letters indicate a significant difference between groups (*P*<0.05)

 Treatments of RA rats were initiated on day 10 post-induction when individual animals had an arthritis index of ≥1. The peak of the arthritis index was recorded on the 15th-day post-RA induction (Figure 4A). All therapies could significantly repress the arthritis index of RA rats 20 days post-induction, compared with untreated RA animals (Figure 4A). The average mean arthritis index was significantly regressed in the RA rats receiving prednisolone or BM-MSC+ES compared with RA rats receiving un-pulsed MSCs during the study period (Figure 4B). Albite, RA rats receiving prednisolone (positive control) showed a faster decrease in the arthritis index compared with RA rats receiving BM-MSC+ES. However, there was no significant difference in the average mean arthritis index between the two groups (Figure 4C).

***Ex vivo*** **results**

**Figure 5:**
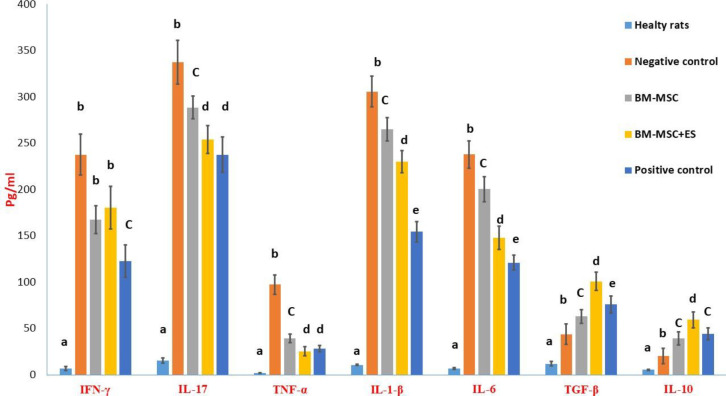
Comparison of cytokine production in the supernatant of spleen cell culture. The weight of all animals was determined before euthanizing animals. Then, the spleens were isolated in the aseptic condition, and the spleen index was calculated for each rat. To determine the proliferation index, spleen cells were pulsed in the presence of collagen II (50 µg/ml) for 72 hr. Different letters indicate a significant difference between groups (*P*<0.05)

Figure 5 illustrates the concentration of pro-inflammatory and anti-inflammatory cytokines in spleen supernatants. The concentrations of TNF-α, IL-1β, IL-6, IFN-γ, and IL-17 were significantly higher in the negative control compared with the healthy rats. The rats in BM-MSCs and BM-MSCs+ES groups showed lower concentrations of these pro-inflammatory cytokines compared with the negative control group (Figure 5). There was no significant difference in interferon-gamma levels between the two groups treated with BM-MSCs or BM-MSCs+ES. Statistically, in reducing the levels of other inflammatory cytokines, treatment with BM-MSCs+ES showed better performance than treatment with BM-MSCs. The RA rats in the positive (prednisolone) group showed the lowest concentration compared with other RA rats for IL-1β, IL-6, and IFN-γ (Figure 5). 

The concentrations of anti-inflammatory cytokines of IL-10 and TGF-β were significantly lower in the normal rats compared with other groups (Figure 5). The data showed that RA rats treated with BM-MSCs+ES could significantly increase the levels of IL-10 and TGF-β compared with the levels of this cytokine gathered from the splenocytes culture of RA rats treated BM-MSCs (Figure 5). Statistically, ES-pulsed BM-MSCs were more successful in increasing anti-inflammatory cytokines than treatment with prednisolone (Figure 5).

**Figure 6:**
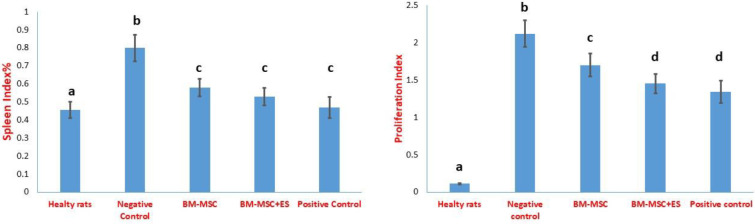
Evaluation of spleen index and splenic lymphocyte proliferative capacity. Spleen cells were pulsed in the presence of collagen II (50 µg/ml) for 72 hr. Then, the supernatant was collected and the production of cytokines was measured by the ELISA method. Findings were presented as mean ±SD. Different letters indicate a significant difference between groups (*P*<0.05)

The splenocyte proliferation and the splenic index increased significantly in the rats with RA groups compared with the healthy control group. The results also showed that the treatment of the rats with BM-MSCs, BM-MSCs+ES, and prednisolone decreased the splenic index compared with the healthy rats (Figure 6). As shown in Figure 6, cell therapy or prednisolone use significantly regressed the proliferative response of the splenic lymphocytes compared with the untreated RA group. Treatment with BM-MSCs+ES or prednisolone resulted in a further reduction in lymphocyte proliferation compared with therapy with BM-MSCs (Figure 6). However, there was no significant difference between the two groups treated with BM-MSCs+ES and prednisolone. 

**Figure 7:**
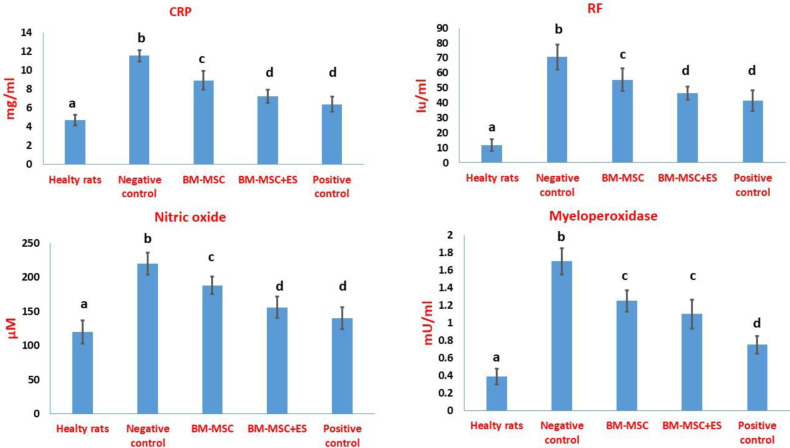
Biochemical changes in the sera of RA rats. At the end of the survey, blood samples were isolated from the hearts of experimental rats under deep anesthesia. The serum samples were used to evaluate CRP, RF, nitric oxide, and myeloperoxidase. Data were reported as mean ±SD. Different letters indicate a significant difference between groups (*P*<0.05)

As shown in Figure 7, the induction of RA increases the serum concentrations of rheumatoid factor and CRP. In RA rats treated with ES-pulsed MSC or MSC, both parameters were reduced significantly. However, the BM-MSCs+ES led to a more significant decline in the serum levels of CRP compared with treatment with un-pulsed BM-MSCs (Figure 7). The ability of ES-pulsed BM-MSCs to reduce CRP and RF was comparable to that of prednisolone (Figure 7). Also, RA induction could significantly mount the levels of MPO and nitric oxide in the sera of rats. The findings showed that RA rats treated with BM-MSCs+ES could significantly reduce the serum levels of nitric oxide more than this factor in RA rats treated BM-MSCs (Figure 7). The ability of ES-pulsed BM-MSCs to decrease the level of nitric oxide was comparable to that of prednisolone (Figure 7). Finally, prednisolone significantly reduced the MPO level more than the other treated groups (Figure 7). 

## Discussion

This survey was conducted to investigate the effects of the estradiol (ES) pulsed bone marrow-derived mesenchymal stem cell (BM-MSC) to ameliorate adjuvant-induced arthritis in Wistar rats. In the first step, the effect of different concentrations of estradiol ES on the immunoregulatory function of MSCs was evaluated. *In vitro* results showed that the least effective concentration of ES that can promote potent anti-inflammatory properties in the MSC population is 100 nM. At this concentration, ES increases the inhibition of the polyclonal T lymphocyte proliferation, production of IDO, IL-10, nitric oxide, TGF-β, and expression of CXCR4 and CCR2 mRNA in the MSC population. Based on *in vitro* results, the RA rats were treated with 2×10^6^ MSCs or ES-pulsed MSCs (100 nM) on day 10 when all animals had developed signs of RA. Results showed that the treatment with BM-MSC pulsed with estradiol led to more relevant and comparable results with prednisolone to reduce the symptoms of RA compared with the treatment with un-pulsed BM-MSC.

Low delivery of mesenchymal stem cells to inflamed tissues is one of the main challenges of using these cells, despite their beneficial modulating power (17). A recent study suggested that treating MSCs with estradiol promotes the migration of cells in cultured MSCs and a cell therapy model of diabetes via adjustment of critical mediators of cell trafficking like hypoxia-inducible factor-1a (HIF-1a) (9). Interestingly, gender could affect the performance of MSCs. Female MSCs stressed by one h hypoxia or LPS (200 ng/ml) significantly produced lower TNF and IL-6 and significantly greater VEGF release than MSCs isolated from male mice (24). Estradiol also improves the differentiation ability and bone regeneration potential of implanted BM-MSCs in a rabbit model of the radial non-union segmental defect (25). 

One of the most critical chemokines and their receptors that affect the migration of BM-MSCs to inflamed areas are stromal cells derived-factor 1 (SDF-1)/ CXCR4 and monocyte chemo-attractant protein (MCP)-1/ CCR2 (9). Estrogens have long been known to exert their functions by turning genes on and off through a multi-step process. Estrogens primarily use two classical nuclear receptors, estrogen receptor α (ERα, Esr1) and ERβ (Esr2), to regulate gene expression. In addition, estrogen receptors change the transcription of genes by interacting with various histone-modifying enzymes and chromatin-remodeling complexes (17, 25). Our results showed that estradiol treatment increased the mRNA expression of CXCR4 and CCR2. However, increasing the concatenation of estradiol from 100 nM to 1000 nM was associated with a decrease in the mRNA expression of these two chemokine receptors in MSCs. Therefore, *in vitro*, conditioning MSCs with 100 nM of estradiol is more effective in increasing the migration potential of stem cells *in vivo*. In 2015, Mirzamahmoudi *et al*. showed that treatment of stem cells with estradiol increased the expression of CXCR4 and CCR2 through induction of hypoxia-inducible factor 1α (HIF-1α) (9).

The main factors responsible for the immunosuppressive and anti-inflammatory benefits of BM-MSCs include surface molecules (like Galectins, PDL1, and HLA-G), anti-inflammatory cytokines (like IL-10 and TGF-β), and secrete some enzymes and molecules (like indole aminepyrrole 2,3-dioxygenase (IDO) and nitric oxide) (20). The current study indicated that increasing the concatenation of estradiol from 100 nM to 1000 nM did not cause a further increase in the anti-proliferative potential of the lymphocytes and other immunoregulatory mediators. Hereupon, the least impressive concentration of estradiol that can induce potent anti-inflammatory phenotype in the MSCs was 100 nM. All this caused MSCs pulsed with 100 nM of estradiol to be used for the following *in vivo* investigations.

The main objectives of this survey were to check out the efficacy of ES-pulsed BM-MSCs to decrease the clinical signs and reset the immune system of RA rats compared with MSc-alone or prednisolone. Despite the potent anti-inflammatory effects of glucocorticoids, they have many side effects. Therefore, using alternative therapies such as mesenchymal stem cells is a logical decision (20). Obtained data in this study revealed that treatment with ES-pulsed BM-MSCs led to a more desirable improvement in the RA severity than using the un-primed BM-MSCs. More importantly, the results of the evaluation of the arthritis index after treatment with ES-pulsed BM-MSCs were similar to those with prednisolone treatment.

Several serum biomarkers have been considered in connection with rheumatoid arthritis or animal models of the disease (20, 26). CRP is an inflammatory factor for RA. The results showed that BM-MSCs with estradiol decreased CRP concentration, confirming the efficiency of BM-MSCs with estradiol in decreasing inflammation. Rheumatoid Factor (RF) belongs to the immunoglobulins family with different isotypes and affinities directed to the Fc portion of IgG. However, it is not specific to RA and found in rheumatic, non-rheumatic conditions, and even healthy adults (27). Regarding RF, the results of this study did not show a significant difference in reducing the level of this factor between the groups treated with BM-MSCs+ES and BM-MSCs. Albite, the BM-MSCs+ES promoted a more significant decrease in the CRP levels compared with therapy with BM-MSCs alone. Furthermore, the potential of BM-MSCs+ES to decline CRP and RF was comparable with that of prednisolone. At the molecular level, CRP is synthesized by the liver in response to pro-inflammatory cytokines like IL-6, IL-1β, and TNF-α (20). As our results showed, treatment with MSCs decreased the level of these pro-inflammatory cytokines. Therefore, reducing the level of CRP is not impossible.

The induction of RA increased the concentration of nitric oxide (NO). Nitrative tissue damage by NO has a close relationship with RA disease. NO induces apoptosis in cartilage and destroys it (4). A study showed a positive correlation between the serum and synovial fluid of patients with RA and NO concentration (28). One of the tip-top biomarkers of inflammatory and oxidative stress in autoimmune diseases like RA is the serum level of MPO (29). Our results also showed that the concentration of MPO was higher in RA rats compared with healthy rats. Similarly, other studies have reported that RA increases the plasma concentration of MPO (30). Our results indicated that the ability of ES-pulsed BM-MSCs to decline the level of nitric oxide was comparable to that of prednisolone. Albite, prednisolone did better than other groups in reducing the MPO level.

The splenic index was significantly higher in the negative control. It means that the spleen increased its size against RA. The spleen has a pivotal role as a reservoir of monocytes/macrophages and lymphocytes activated during inflammation and produces cytokines and chemokines (31). The results of the spleen index confirm the relevant results of the treatment protocols. The capability of medication to reduce lymphocyte proliferation restricts the number of potentially pathologic T cells in RA. Both prednisolone and MSCs therapy possess anti-proliferative effects (20). According to the results of this study, the treatment of BM-MSCs with estradiol increased their immunosuppressive properties, so that the strength will be comparable to prednisolone.

The induction of RA increased the concentration of pro-inflammatory cytokines while decreasing the concentration of anti-inflammatory cytokines. Pro-inflammatory cytokines are potential therapeutic targets for RA, and cytokines promote inflammatory responses in arthritic joints and synovial tissues (3). TNF-α plays a pivotal role in the inflammatory and immunological responses to RA development and it is generally known as a promising target for an anti-RA drug. IL-1β and IL-6 are critical pro-inflammatory cytokines involved in the development of RA (1, 3, 26). The Th17 and Th1 cells are critical players in RA disease. IL-17 and IFN-γ possess potent pro-inflammatory properties and are the main factors for Th17 and Th1-mediated immunopathology, respectively (32). The inflammation process is significantly controlled and balanced by mediators that induce and sustain inflammation and mediators that shut down the process and are called anti-inflammatory cytokines such as IL-10 and TGF-β (1). Based on our results, prednisolone was more successful in reducing inflammatory cytokines than treatment with ES-pulsed BM-MSCs. Conversely, ES-pulsed BM-MSCs were more successful in increasing anti-inflammatory cytokines than pharmacotherapy with prednisolone. Therefore, it can be assumed that prednisolone acts by paralyzing immune responses, while BM-MSCs act more by amplifying anti-inflammatory responses. This may be another advantage of using estradiol-treated BM-MSCs to control RA.

**Table 1:** Primers used to evaluate the expression of CCR2 and CXCR4 chemokine receptors

**Gene**	**Forward**	**Reverse**
***CCR2***	_5ʹ-TGATCCTGCCCCTACTTGTCAT-3ʹ_	_5ʹ-ATGGCCTGGTCTAAGTGCATGT-3ʹ_
***CXCR4***	_5ʹ-GGAAGGAACTGAACGCTCCAGAA-3ʹ_	_5ʹ-GAAACCACACAGCACAACCAAAC-3ʹ_
***β-actin***	_5ʹ-TGTCCACCTTCCAGCAGATGT-3_	_5ʹ AGCTCAGTAACAGTCCGCCTAGA-3ʹ_

## Conclusion

Treatment of MSCs with estradiol increased the regulatory potential of these cells compared with untreated MSCs. The results of the *in vivo *study showed a better improvement in RA signs of rats that received ES-pulsed MSCs compared with the symptoms of RA rats which received MSCs. The clinical results of treatment with ES-pulsed MSCs were comparable to treatment with prednisolone. Due to the excellent potential of ES-pulsed BM-MSCs in reducing the symptoms of the disease, this approach may be a helpful strategy for controlling RA. The main limitation of the current study is conducting a study on rats, and the results cannot be used for other animals and humans. Further studies are required to show the efficiency of BM-MSCs treated with estradiol in humans.

## Authors’ Contributions

SMAF designed the experiments; MJ performed experiments and collected data; SMAF and NAA supervised, directed, and managed the study; MJ, SMAF, and NAA finally approved the version to be published.

## Conflicts of Interest

None.
